# Evaluation of Speakers at CME: Cosmecon 2006, An International Conference on Ageing and Anti-ageing

**DOI:** 10.4103/0974-2077.44170

**Published:** 2008

**Authors:** Vijayashankar MR

**Affiliations:** *Department of Dermatology, Sri Siddhartha Medical College, Tumkur, Karnataka, India*

**Keywords:** CME in dermatology, evaluation of speakers, session evaluation

## Abstract

**Aim:**

To determine what constitutes effective or ineffective lecturing in dermatological conferences and also the utility of a scientific programme for the dermatologists.

**Methods and Materials:**

Evaluation forms were circulated to delegates attending the Cosmecon conference on ageing and anti-ageing, held in July 2006 at Bangalore. Feedback from the delegates in the form of completed evaluation forms of individual speakers and each session of the 3-day conference Cosmecon (including the live workshop on aesthetic and surgical procedures) were studied. Comments were analysed with the help of a biostatistician to determine the positive and negative responses.

**Results:**

On day 1 of the conference, workshop included 14 procedures by different specialists and on the second and third days of the conference, there were a total of 10 sessions, with five on each day. Evaluation forms were handed out to 440 delegates on day 1 and 600 delegates on days 2 and 3. Fifty-five speakers were evaluated by an average 56 delegates out of 440 delegates on day 1 and 600 delegates on the second and third days. The delegate response to completing the evaluation form was poor. Only about 25% of the delegates completed the feedback forms. However, the feedback did give some insight to the scientific programme, on both positive and negative aspects. Most delegates stated that they benefited from the presentations. The main negative response was lack of opportunity to ask questions after a lecture. The main positive comment was that the time keeping in the conference was very good.

**Conclusion:**

The response of the delegates in providing feedback was poor. Efforts have to be made to educate and encourage delegates to complete the feedback forms. Systematic review of the speakers would provide information to design future CME programmes effectively and to incorporate improvements for effective lecturing and to avoid ineffective lectures. The CME evaluation can also help the organizers to provide training to presenters and to monitor performance.

## INTRODUCTION

CME programme has become very important in the advancement/refreshing of knowledge in the latest developments in the field of dermatology. Different formats such as lectures, hands-on workshops and live demonstrations with interactive sessions coupled with electronic and digital presentation of lectures are some of the formats used in the scientific programs. Although an increasing number of CMEs are being conducted in India, no attempt has been made, to the best of our knowledge, to evaluate the outcome of such conferences. Pubmed search did not reveal such detailed study with respect to the speciality of dermatology. This, therefore, is the first such attempt, to the best of our knowledge and has been carried out at an International conference – Cosmecon 2006 – a conference on ageing and anti-ageing, held at Bangalore, India. This article makes an attempt to analyse delegate feedback regarding the evaluation of speakers and to learn about the effectiveness of this conference.

## METHODS AND MATERIALS

Cosmecon 2006 was held at the Gnanabharati auditorium of Bangalore University from 14^th^ to 16^th^ July, 2006. It was sponsored by Bangalore Dermatological Society and was accredited by the American Academy of Continuing Medical Education. The course was accredited for 27 ½ h of CME credits.

Eleven sessions were programmed in this 3-day conference. On day 1, the entire day was a single workshop, with 15 speakers performing live different aesthetic and dermatosurgical procedures. On days 2 and 3, there were a total of 10 sessions, consisting of didactic lectures, each session with four speakers.

All speakers used digital presentations. Delegates were given booklets containing tear-off evaluation forms [Tables 1 and 2]. The format in the feedback forms originally used by Collins J, Mullan BF, Holbert JM was adapted and modified to suit this conference.[1] Separate analysis was carried out for sessions and for individual speakers.

**Table 1:** Session evaluation points

Participants respond to items 1-10 with one of the following:												
1 (Strongly Agree), 2 (Agree), 3 (Disagree), 4 (Strongly Disagree)												
Date	14-Jul-06	15-Jul-06					16-Jul-06					
Session	--	1	2	3	4	5	1	2	3	4	5	6
Evaluation points												
Presentation followed the prescription in the programme												
Presentation began on time and was well placed												
The faculty communicated clearly and effectively												
The faculty allowed enough time for questions and answers												
The information presented was clearly relevant												
The content was objective and free of commercial bias												
The content was scientifically rigorous												
The audiovisual aids were effective												
I expect to make changes in practice from what I learned												
I improved my knowledge as a result of this presentation												

Please tell us what topic you think would like to have covered in future meetings.

And also tell us your overall experience of the meet in a few words.

Please hand over this form duly filled to the conference official at the reception.

Your Name:

COSMECON Registration #

IADVL Membership #

**Table 2:** Delegate feedback forms for the conference program

COSMECON 2006 - Audience response sheet				
Time	Topic	Your response		

		Question 1	Question 2	Question 3
**Date: 14 July 2006**	**Time: 8 AM - 5 PM**			
8.30 AM - 9.00 AM	Chemical peel I			
9.00 AM - 9.40 AM	Mesotherapy			
9.40 AM -10.00 AM	Targeted ultrasonic lipolysis			
10.00 AM - 10.30 AM	Laser peel			
10.30 AM - 11.15 AM	Non ablative optical skin rejuvenation			
11.15 AM - 12.30 PM	Thread lift			
				
12.30 PM - 1.00 PM	Erbium glass laser rejuvenation			
1.00 PM - 1.45 PM	Guided video presentation			
	Hair transplant			
	Liposuction			
1.45 PM - 2.45 PM	Botox			
2.45 PM - 3.45 PM	Fillers			
3.45 PM - 4.15 PM	Intense pulsed light photorejuvanation			
4.15 PM - 4.45 PM	Chemical Peel II			
4.45 PM - 5.15 PM	Radiolift			
	Gray hair reduction			
Date: 15 July 2006	Time: 9 AM - 5 PM			
Session I - 9.00 AM - 11.05 AM - Aging in Indian skin				
9.00 AM - 9.10 AM	Clinical features			
9.10 AM - 9.25 AM	Mechanisms of aging			
9.25 AM - 9.35 AM	Psychological aspects			
9.35 AM - 9.45 AM	Aging ungracefully			
10.45 AM - 11.05 AM	Anti-aging treatments - Where are we heading?			
Session II - 11.05 AM - 11.45 AM - Strategies for anti aging therapies				
11.05 AM - 11.15 AM	Cosmoceuticals in antiaging			
11.15 AM - 11.25 AM	Pharmaceuticals in antiaging			
11.25 AM - 11.35 AM	Sun and aging			
11.35 AM - 11.50 AM	Nonablative rejuvenation			
Session III - 12.00 PM - 1.00 PM - Facial rejuvenation				
12.00 PM - 1.15 PM	Mesotherapy, pros and cons			
12.15 PM - 12.30 PM	Fillers in aging skin			
12.30 PM - 12.40 PM	Newer peels			
12.40 PM - 12.55 PM	IPL in facial rejuvenation			
2.00 PM - 2.20 PM	Fractional photothermolysis			
Session IV - 2.20 PM - 3.30 PM - How I manage				
2.20 PM - 2.35 PM	Acne scars & sebaceous hyperplasia			
2.35 PM - 2.45 PM	Nasolabial furrow			
2.45 PM - 2.55 PM	Melasma			
2.55 PM - 3.05 PM	Aging nails			
3.05 PM - 3.15 PM	Pigmented lesions in aging skin			
4.00 PM - 4.15 PM	Changing trends in facial rejuvenation			
Session V - 4.15 PM - 5.00 PM - Indian Experience				
				
Date: 16 July 2006	Time: 8.30 AM - 5.00 PM			
Session I - 8.30 AM - 9.30 AM - Geriatric dermatology				
8.30 AM - 8.40 AM	Generalized pruritus of the elderly			
8.40 AM - 8.50 AM	Neck rejuvenation			
8.50 AM - 9.05 AM	Sclerotherapy			
9.05 AM - 9.20 AM	Side effects of cosmetics			
Session II - 9.30 AM - 10.10 AM - Practical problems and dilemmas				
9.30 AM - 9.40 AM	How to set up an antiaging skin clinic			
9.40 AM - 9.50 AM	Ethics & anti-aging			
9.50 AM - 10.05 AM	Marketing strategies of aesthetic techniques			
Session III - 10.30 AM - 11.15 AM - What is New?				
10.30 AM - 10.45 AM	Erbium glass laser			
10.45 AM - 11.00 AM	Stem cells and aging			
11.00 AM - 11.15 AM	Thread lifts			
Session IV - 11.15 AM - 12.00 PM - Hair				
11.15 AM - 11.30 AM	Treatment of hair loss			
11.30 AM - 11.40 AM	Grey hairs			
Session V - 12.00 PM - 1.00 PM - FAT				
12.00 PM - 12.15 PM	Liposuction			
12.14 PM - 12.25 PM	Laser lipolysis			
12.25 PM - 12.40PM	Ultrasonic lipolysis			
Session VI - 3.00 PM - 5.00 PM - Scientific session for general practioners				
3.15 PM - 3.25 PM	Aging changes in skin			
3.25 PM - 3.35 PM	Acne scars			
3.35 PM - 3.45 PM	Melasma.			
3.45 PM - 3.55 PM	Hair loss			
3.55 PM - 4.05 PM	Lasers in dermatology			
4.05 PM - 4.15 PM	Hair removal			
4.15 PM - 4.25 PM	Pruritus in the elderly			

Thank You for attending the programmes. Please take a few minutes to respond based on the questions below by marking the appropriate rating.

1. Did you find this programme worthwhile and educational?

2. Rate the speakers presentation style and quality

3. Did the speaker satisfactorily address the program description?

Rating scale: E = Excellent, VG = Very good, G=Good, F=Fair, P=Poor

The individual speakers were evaluated with their names and topics listed for rating as per the scheme in appendix. Additional comments were invited on a separate space provided. The forms circulated asked for feedback on specific aspects such as affectivity, punctuality, objectivity, presence of commercial bias, scientific value and effectiveness of audiovisual aids.

As the total number of feedback forms received was small and therefore the sample obtained was not significant to perform a detailed statistical analysis, a subjective evaluation was performed using the data available. In some cases, the data were extrapolated to a higher number for analysis and to avoid small fractions.

## RESULTS

Only about 25% of the delegates completed the feedback forms. It was also interesting to note that many feedback forms had been filled poorly. There were forms where comments were illegible.

Analysis of the session evaluation forms [Table 3] on day 1 of the workshop day showed that the program evoked a generally positive response. Most delegates agreed that the program began on time and was well placed. Communication of the faculty was also well appreciated. Over 50% of the delegates felt that their knowledge had improved as a result of the program. However, there were many negatives also. The audiovisual aids were regarded as ineffective. Thirty per cent of the delegates felt that there was commercial bias and that scientific verve was suboptimal. Except for one speaker, all others got a positive response.

**Table 3:** Results of session evaluation by delegates

Session evaluation form items	Strongly agree	Agree	Disagree
Presentation followed the description in the program	58	28	14
Presentation began on time and was well placed	58	25	14
The faculty communicated clearly and effectively	28	44	28
The faculty allowed enough time for questions and answers	25	44	28
The information presented was clinically relevant	14	44	42
The content was objective and free of commercial bias	12	57	31
The content was scientifically rigorous	12	58	30
The audiovisual aids were effective.	43	14	43
I expect to make changes in practice from what I learned	43	29	28
I improved my knowledge as a result of this presentation	57	14	29

All in percentages

On day 2, sessions 1 and 2 got a favourable and positive response in contrast to sessions 3–5 which received some negative response. The most common negative response was that the faculty did not allow enough time for interaction with audience. The most positive response was to the speakers in the first three sessions.

On day 3, the response was equivocal, with most delegates indicating responses as strongly disagree to strongly agree. In this session, a small percentage (10%) did not agree that the sessions improved their knowledge. Most individual speakers got a good response, except those who spoke on *laser lipolysis, aging nails and non-ablative rejuvenation*.

There were many post-script comments, both positive and negative [Table 4], which provided a better insight into the efficiency and effectiveness of the program. Many respondents seemed to fill the forms mechanically without applying their mind, which was reflected in some observations as follows:

**Table 4:** Post-script comments by the delegates

Negative comments	Positive comments
Audiovisual failure	The meeting was excellent, well organized and had depth
Time allotted was inadequate to many speakers	The time keeping was very good
Speakers used up their time in history and introduction (Newer peels – only 3 min for the topic)	Food was generally very good
Repetition of topics	The meeting was a grand success
Foreign delegates should not have been interrupted by the chair	
One person was hogging the lime light during Q and A sessions	
Dermatological problems were ignored	
Some chairpersons were not allowing the speakers to interact	
There was commercial bias (fillers in ageing)	
Q and A session was inadequate	
Audience in the back seats were not allowed free access to ask questions	
Same persons were asking questions	
No. of questions per individual must be limited to one only

- Delegates gave a feedback on a lecture that was not delivered (sclerotherapy)!
- Respondents seemed to have filled the form in one sitting hurriedly, even illegibly, and not after each lecture.
- Some delegates had marked the appendix also with responses.
- Some delegates had used the response sheet for taking notes.
- Some wanted more on vitiligo (which did not form a part of the anti-ageing spectrum of topics).
- Some wanted more discussion on medical indemnity and damage claims.
- Some wanted more on dermatosurgery.

Similar observation as above have been previously reported in other meetings as well.[1]

Some delegates who did not attend or came late for the sessions also filled up the entire feedback form. This was evident by a response where the delegate emphasized on having a session on “Medico legal aspects on laser”, unaware of the fact that the topic was already covered in the conference! Some delegates suggested few inclusions into the curriculum, viz iontophoresis.

## DISCUSSION

Though lectures are the most popular method of imparting knowledge, they are regarded as poor methods to promote knowledge, and lack of involvement of delegates is a serious limitation of this format of knowledge dissemination. Analysis of the program in the light of delegate’s feedback gives an excellent opportunity to improvise the same and saves huge investment in the form of time, manpower and finance. The information obtained by us provides useful, although not comprehensive, information about the value of scientific programs.

This is a novel exercise in the dermatology conference in India and one would have expected a more enthusiastic response from the delegates. However, only 25% of the delegates filled the feedback forms. This could be either because the delegates were not aware of the impact of their feedback or because of their disinterest in this exercise.

Characteristics of an effective lecture presentation have been well reported by Gelula.[2] It calls for more than just offering ideas and data to an audience. It calls for direct contact with the audience, effective use of language, capability to use limited time effectively and the ability to be entertaining. The same has been emphasized in their articles by Gigliotti, van Dokkum and Copeland.[3–5]

There were several limitations in this study as mentioned earlier and yet, it provided interesting findings both with respect to the speakers’ evaluation as well as the delegates’ feedback. Thus, though the effort was not comprehensive and rewarding enough to allow statistical analysis, it did show some interesting data and proved to be an earnest attempt to evaluate speakers/CME program organizers. We hope this will initiate similar analysis of other conferences in future, evaluating and designing the CME program to render it interesting, informative and entertaining at the same time.

The data of this study showed that greater participation by the delegates is needed. Further interaction for content presentation and interview with participants is required in future for assessment. The evaluation sheets could be redesigned and simplified to suit the dermatologist’s requirements and a systematic approach to collect data is required.
